# *REL2*, A Gene Encoding An Unknown Function Protein which Contains DUF630 and DUF632 Domains Controls Leaf Rolling in Rice

**DOI:** 10.1186/s12284-016-0105-6

**Published:** 2016-07-29

**Authors:** Shuai-Qi Yang, Wen-Qiang Li, Hai Miao, Peng-Fei Gan, Lei Qiao, Yan-Li Chang, Chun-Hai Shi, Kun-Ming Chen

**Affiliations:** 1 State Key Laboratory of Crop Stress Biology in Arid Areas, College of Life Sciences, Northwest A&F University, Yangling, 712100 Shaanxi People’s Republic of China; 2 Department of Agronomy, College of Agriculture and Biotechnology, Zhejiang University, Hangzhou, 310058 Zhejiang People’s Republic of China

**Keywords:** Rice (*Oryza sativa L.*), Rolling and erect leaves, *REL2*, DUF domains, Bulliform cells

## Abstract

**Background:**

Rice leaves are important energy source for the whole plant. An optimal structure will be beneficial for rice leaves to capture light energy and exchange gas, thus increasing the yield of rice. Moderate leaf rolling and relatively erect plant architecture may contribute to high yield of rice, but the relevant molecular mechanism remains unclear.

**Results:**

In this study, we identified and characterized a rolling and erect leaf mutant in rice and named it as *rel2*. Histological analysis showed that the *rel2* mutant has increased number of bulliform cells and reduced size of middle bulliform cells. We firstly mapped *REL2* to a 35-kb physical region of chromosome 10 by map-based cloning strategy. Further analysis revealed that *REL2* encodes a protein containing DUF630 and DUF632 domains. In *rel2* mutant, the mutation of two nucleotide substitutions in DUF630 domain led to the loss-of-function of *REL2* locus and the function of *REL2* could be confirmed by complementary expression of *REL2* in *rel2* mutant. Further studies showed that REL2 protein is mainly distributed along the plasma membrane of cells and the *REL2* gene is relatively higher expressed in younger leaves of rice. The results from quantitative RT-PCR analysis indicated that *REL2* functioning in the leaf shape formation might have functional linkage with many genes associated with the bulliform cells development, auxin synthesis and transport, etc.

**Conclusions:**

REL2 is the DUF domains contained protein which involves in the control of leaf rolling in rice. It is the plasma membrane localization and its functions in the control of leaf morphology might involve in multiple biological processes such as bulliform cell development and auxin synthesis and transport.

**Electronic supplementary material:**

The online version of this article (doi:10.1186/s12284-016-0105-6) contains supplementary material, which is available to authorized users.

## Background

Plant leaves are lateral organs and derived from the peripheral zone of the shoot apical meristem (SAM) (Sussex 1995; Bowman et al. 2002). As the leaf grows away from the meristem, its shape is decided by growing in three different axes, which are proximal-distal, abaxial-adaxial and medial-lateral. Among these three axes, the adaxial-abaxial axis is the foundation of the subsequent asymmetric growth of the leaf and lamina expansion (Waites and Hudson 1995; McConnell and Barton 1998; Moon and Hake 2011). Rice is one of the most important crops in the world and how to increase grain yield has become a focus on rice research. Appropriate leaf shape is an important characteristic to construct the super-high-yield hybrid rice, which means long, narrow, V-shaped (rolling) and thick leaves of the top three leaves (Yuan 1997). Moderate leaf rolling will be beneficial to the erectness of leaves, thus minimizing shadowing among leaves, which contributes to improving photosynthetic efficiency and grain yield (Zhang et al. 2009; Xiang et al. 2012). In order to achieve a breakthrough of rice yield, isolation and identification of rolling leaf genes is a necessary way to construct ideal plant architecture with moderate leaf rolling (Price et al. 1997).

The genetic mechanism behind leaf in adaxial-abaxial axis development is relatively clear in *Arabidopsis thaliana*. Many mutants have been investigated and the results illustrated that HOMEODOMAIN-LEUCINE ZIPPERIII (HD-ZIPIII), KANADI and the small RNA pathway play significant roles to determine the shape in adaxial-abaxial growth (for a review Moon and Hake 2011). However, the mechanism about leaf in adaxial-abaxial axis development in rice is still in exploration and not so clear. Generally, there are two types of leaf rolling in rice, adaxial rolling and abaxial rolling. To date, more than fourteen rolling leaf (*rl*) mutants in rice were isolated and mapped genetically. Among these mutants, six recessive genes (*rl1-rl6*) were mapped on rice chromosomes 1, 4, 12, 3, 1 and 7 by morphological markers, respectively (Khush and Kinoshita 1991). *rl7* to *rl12*, *rl14* and *rl(t)* were located on chromosomes 2 (*rl(t)*, Shao et al. 2005a), 5 (*rl(7)*, Li et al. 1998; *rl(8)*, Shao et al. 2005b), 7 (*rl11*, Shi et al. 2009), 9 (*rl9*, Yan et al. 2008; *rl10*, Luo et al. 2007), and 10 (*rl12*, Luo et al. 2009; *rl14*, Fang et al. 2012), respectively. However, only *rl9* and *rl14* were cloned and analyzed in detail.

There are some transcription factors which were demonstrated being involved in controlling leaf rolling. *RL9*, an orthologue of *Arabidopsis KANADIs*, encodes a Glycoprotein A Repetitions Predominant (GARP) protein and mainly expresses in roots, leaves and flowers. Subcellular localization indicated that *RL9* acts as a putative transcription factor due to localizing in the nucleus (Yan et al. 2008). *OsMYB103L* encodes an R2R3-MYB transcription factor and is localized in the nucleus with transactivation activity. Over-expression of *OsMYB103L* results in a rolled leaf phenotype. In contrast, knockdown of this gene leads to reduced cellulose content and mechanical strength in leaves (Yang et al. 2014). *OsZHD1* is a zinc finger homeodomain class homeobox transcription factor, which exhibits a constitutive expression pattern in wild type (WT) and accumulates in the developing leaves and panicles. Over-expression of *OsZHD1* and its homolog *OsZHD2* causes the abaxial leaf curling with increased number and abnormal arrangement of bulliform cells (Xu et al. 2014). *SHALLOT-LIKE 1* (*SLL1*) encodes a SHAQKYF class MYB family transcription factor which belongs to the KANADI family and *sll1* mutant displays extremely incurved leaves because of the defective development of sclerenchymatous cells on the abaxial side. Further study indicated that *SLL1* deficiency leads to abnormal programmed cell death of abaxial mesophyll cells and suppresses the development of abaxial features. However, phloem development on the abaxial side is stimulated and bulliform cell and sclerenchyma development on the adaxial side are both suppressed by enhanced *SLL1* expression (Zhang et al. 2009).

In addition, some other genes found in rice mutant also play key roles in controlling leaf rolling. For example, *narrow and rolled leaf 1* (*nrl1*) encoding a cellulose synthase-like D4 protein is characterized by a phenotype of narrow and rolled leaves (Wu et al. 2010). *ADAXIALIZED LEAF 1* (*ADL1)* encodes a plant-specific calpain-like cysteine proteinase orthologous to maize *DEFECTIVE KERNEL1* and *adl1* mutant shows abaxially rolled leaves (Hibara et al. 2009). *OsAGO7* including the PAZ (Piwi/Argonaute/Zwille) and Piwi conserved domains belongs to the Argonaute (Ago) family and could be orthologous to the *Arabidopsis thaliana Ago7* gene. Over- expression of this gene leads to upward curling of the leaf blade (Shi et al. 2007). SEMI-ROLLED LEAF 1 (SRL1) is located at plasma membrane and predicted to be a putative glycosylphosphatidylinositol-anchored protein. *srl1* mutant exhibits adaxially rolled leaves due to the increased number of bulliform cells at the adaxial cell layers. Further study showed that *SRL1* can inhibit the formation of bulliform cells by regulating the expression of genes encoding vacuolar H^+^-ATPase subunits and H^+^-pyrophosphatase negatively (Xiang et al. 2012). *LEAF INCLINATION 2* (*LC2*) encodes a vernalization insensitive 3-like protein which plays an important role in regulating leaf inclination and mediating hormone effects and *lc2* mutants have enlarged leaf angles and abaxial leaf curling (Zhao et al. 2010). *ABAXIALLY CURLED LEAF 1* (*ACL1*) encodes a protein containing unknown conserved functional domains and over-expression of *ACL1* and its homolog *ACL2* induces abaxial leaf curling (Li et al. 2010). Most recently, a new gene called *SRL2* has been identified and cloned. This gene encodes a novel plant-specific protein of unknown biochemical function. *srl2* mutant has incurved leaves due to the presence of defective sclerenchymatous cells on the abaxial side of the leaf and displays narrow leaves and reduced plant height. Double mutant analysis indicated that *SRL2* and *SLL1/RL9* function in different ways to regulate abaxial-side leaf development (Liu et al. 2016). At last, *ROLLED and ERECT LEAF 1* (*REL1*) encoding a novel no known protein is related to the coordination of brassinosteroid (BR) signaling transduction. Also, *rel1* mutant displays rolled and erect leaf and over-expression of *REL1* in WT causes a phenotype similarity to that of the dominant *rel1* mutant. However, down-regulation of the *REL1* gene in the *rel1* mutant restores the mutant phenotype (Chen et al. 2015). Considering the similar phenotype as to *rel1*, we named our mutant as *rel2*.

In eukaryotes, a number of gene families that encode functionally uncharacterized protein were identified as domain of unknown function (DUF) and evolutionary conservation suggested that many DUFs have important functions (Goodacre et al. 2014; Wang et al. 2014). Some “Domains of Unknown Function” (DUFs) or “Uncharacterized Protein Families” (UPFs) in Pfam database have been analyzed for both structure and function by computational structural genomics approach (Betman et al. 2010; Goodacre et al. 2014; Mudgal et al. 2015). However, only few of them were determined in detail.

In the present study, we identified a rolling and erect leaf mutant and isolated the control gene by using map-based cloning strategy. Then, the subcellular localization and expression profiles of the control gene were investigated. The results obtained here provide a new important reference for understanding the mechanism of rolling leaf blades in rice.

## Results

### Phenotypes of *rel2*

**Fig. 1:**
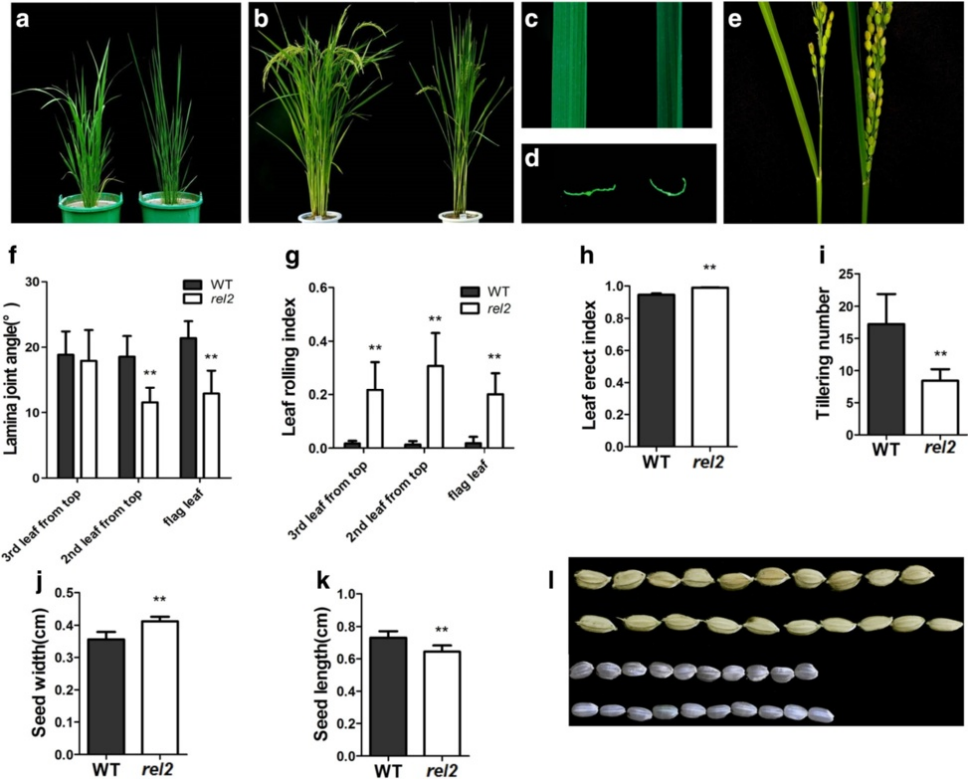
Characterization of WT and *rel2* mutant. **a**, **b** Morphology of WT (*left*) and *rel2* mutant (*right*) at tillering stage and heading stage, respectively. **c** Morphology of second leaf from the top of WT (*left*) and *rel2* mutant (*right*) at heading stage. **d** Transverse section of the middle part of the second leaf from the top of WT (*left*) and *rel2* mutant (*right*) at heading stage. **e** The display of angle between panicle and the flag leaf of WT (*left*) and *rel2* mutant (*right*). **f**-**k** Statistical analysis of lamina joint angle, LRI, LEI, tillering number, seed length and seed width. At least 20 samples of WT and *rel2* mutant were measured for each. The values are shown as mean ± SD. Double asterisk (**) indicates that the difference between the WT and *rel2* is statistically significant at *P* < 0.01. **l** Morphology of grains with husk and without husk of WT (*down*) and *rel2* mutant (*up*) in every comparison

**Fig. 2:**
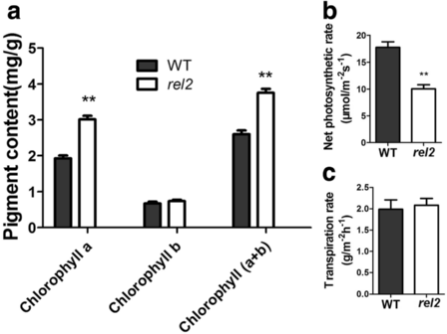
Characterization of pigment content and photosynthetic rate of *rel2* mutant. **a** Pigment contents of third leaf from the top at heading stage. **b**, **c** Comparison of net photosynthetic rate and transpiration rate between WT and *rel2* mutant. Pigment contents data are mean ± SD (*n* = 3). Net photosynthetic rate and transpiration rate data are mean ± SD (*n* ≥ 15). *Double asterisk* (* *) indicates that the difference between the WT and *rel2* is statistically significant at *P* < 0.01

The mutant was isolated from M_2_ generation of rice cultivar Nipponbare (*Oryza sativa* L. *japonica*) by mutagenesis with ethyl methanesulfonate (EMS), and was named as *rolled anderectleaf 2* (*rel2*) mainly based on its abnormal phenotypes of leaves. Compared with the WT, the *rel2* mutant plants have adaxially rolling and erect leaves which lead to erect architecture of rice plants. During the early seedling stage, the phenotype of *rel2* mutant is becoming different from that of WT. As the rice grows, significant phenotypic differences could be distinguished from the two type plants. Figure 1a, b shows the gross morphology of WT and *rel2* mutant at tillering stage and heading stage, respectively. In contrast to the WT, all leaves of *rel2* exhibit adaxial rolling (Fig. 1c, d). The *rel2* mutant also shows erect growth of two top leaves, owing to reduced lamina joint angle (Fig. 1e, f). The leaf rolling index (LRI) and the leaf erect index (LEI) are significantly increased in *rel2* mutant (Fig. 1g, h). In addition, the *rel2* mutant also exhibits other abnormal phenotypes, such as reduced tillering number, round grains, reduced number of grains per main panicle (Fig. 1i to l, Table 1). Since all the leaf blades in *rel2* mutant plants display darker green leaf phenotype during almost all the growth and development stages, the pigment contents in leaves were also investigated here. The results showed that chlorophyll a (Chl a) and chlorophyll b (Chl b) contents in mutant leaves are obviously higher than those in WT (Fig. 2a). Even more, the mutant’s net photosynthetic rate is decreased whereas its transpiration rate is slightly increased compared with the WT (Fig. 2b, c). Also the number of adventitious roots is significantly less than that of WT. Shorter adventitious roots can also be observed in the *rel2* mutant plants (Additional file 1: Figure S1). These observations indicated that the *rel2* mutant has multiple morphological defects.

**Table 1:** Comparison differences of some key agronomic traits between WT and *rel2* mutant

Traits	NIP	*rel2*
The number of grains per panicle	128 ± 18	58 ± 6^* *^
Fertility rate (%)	88.3 ± 3.9	69.7 ± 6.2^* *^
1,000-grain weight (g)	24.8 ± 0.2	26.7 ± 0.4^* *^
Plant height (cm)	94.5 ± 4.0	72.8 ± 2.7^* *^
Panicle length (cm)	22.0 ± 1.9	16.9 ± 1.5^* *^
Flag leaf length (cm)	29.7 ± 4.0	34.8 ± 6.3^* *^
Second leaf length (cm)	38.3 ± 4.8	46.8 ± 4.9^* *^
Third leaf length (cm)	37.3 ± 4.8	39.1 ± 4.1

The values were shown as mean ± SD (*n* ≥ 20). Double asterisk (* *) indicates that the difference between the WT and *rel2* is statistically significant at *P* < 0.01

### Abnormal Bulliform Cell Number, Size and Arrangement in *rel2* Leaf Blades

**Fig. 3:**
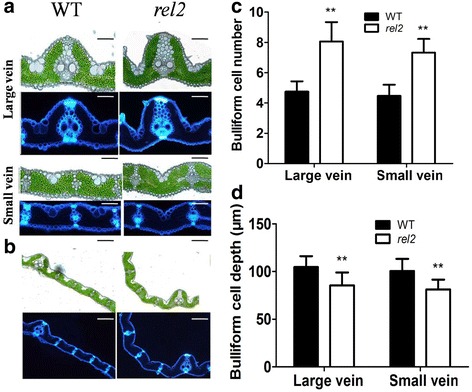
Histological analysis of the leaf blades between WT and *rel2* mutant. **a** Transverse section of large vein and small vein at the *middle* of the third leaf from the *top* at heading stage, Bars = 100 μm. **b** Transverse section of leaf blades at the *middle* of the third leaf from the *top* at heading stage, Bars = 200 μm. **c** Statistics analysis of the number of bulliform cells besides large veins and small veins between WT and *rel2* mutant, data are mean ± SD (*n* ≥ 10). **d** Statistics analysis of the depth of bulliform cells on the sides of large veins and small veins between WT and *rel2* mutant, data are mean ± SD (*n* ≥ 10). *Double asterisk* (* *) indicates that the difference between the WT and *rel2* is statistically significant at *P* < 0.01

Considering the important roles of bulliform cells in determining leaf flatting in rice (Xiang et al. 2012), an analysis of free-hand cross section was carried out in present study. Compared with that of WT, more bulliform cells are observed on adaxial epidermis of *rel2* leaf blades (Fig. 3a, b). In the WT leaf blades, bulliform cells are arranged in groups of 5 ± 1 cells, with the middle cells larger than those on either side; whereas in the *rel2* mutant, bulliform cells are arranged in groups of 8 ± 1 cells, with middle cells as small as those of other cells (Fig. 3a, b). Statistical analysis revealed a significant increase of bulliform cell number in *rel2* mutant (Fig. 3c). Moreover, we found that the bulliform cells are arranged with “U” shape in WT, whereas the bulliform cells of *rel2* mutant are arranged with “V” shape (Fig. 3a, b). The depth of bulliform cells is also decreased in *rel2* leaf blade as compared to the WT (Fig. 3d). These results indicated that the rolling leaf phenotype should result from the reduced size of middle bulliform cells and “V” shape arrangement of bulliform cell region.

### Map-based Cloning of *REL2* Gene

In order to isolate *REL2* by map-based cloning strategy, a genetic population was constructed by crossing *rel2* mutant with an *indica* rice cultivar, Kasalath. All F_1_ hybrids derived from the cross showed normal phenotype, similar to that of Kasalath. In the F_2_ population, we planted 200 individuals, and identified 142 normal plants and 58 *rel2* plants, respectively. The segregation ratio is just fitted to 3:1 (*χ*^2^ = 0.812 < χ^2^_0.05_ = 2.85), indicating that the *rel2* phenotype is controlled by a single recessive nuclear gene.

**Fig. 4:**
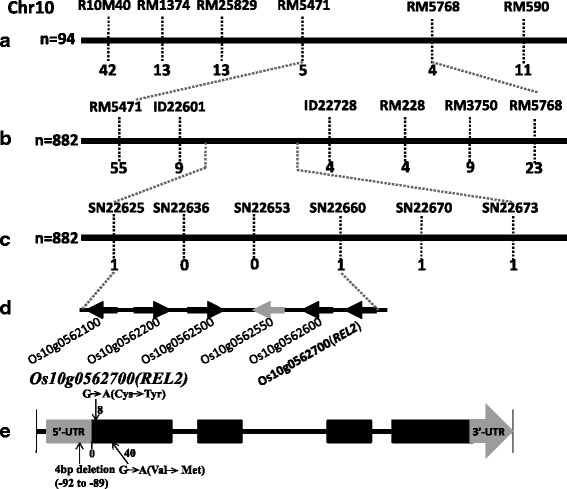
Map-based cloning of *REL2.* **a**-**c** Primary mapping of *REL2* (**a**) and fine mapping of *REL2* (**b**, **c**). The genetic linkage map is derived from 94 F_2_ mutant individuals for primary mapping and 882 F_2_ mutant individuals for fine mapping. Markers’ names are above the *vertical lines* and the number of recombinants is displayed under the *vertical lines*. **d** The six candidate genes between SN22626 and SN22660. **e** Gene structure and mutant sites of candidate gene *Os10g0562700 (REL2)*. *Black boxes* indicate the exons and *grey boxes* represent untranslated region (UTR). The *rel2* mutant has a 4-bp (GGAG) deletion in 5’-UTR and two point mutations (G to A and G to A) in the first exon

**Fig. 5:**
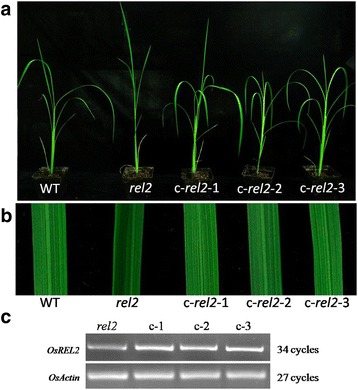
Complementary experiment of *rel2* mutant. **a** WT and *rel2* mutant and T_0_ transgenic plants c-*rel2*-1, c-*rel2*-2 and c-*rel2*-3. **b** Comparison of morphology of leaf blades among WT, *rel2* mutant, c-*rel2*-1, c-*rel2*-2 and c-*rel2*-3. The leaf pieces are derived from the same sites at middle of leaf blades. **c** RT-PCR of *REL2* in *rel2* mutant and three independent complementary lines c-*rel2*-1, c-*rel2*-2 and c-*rel2*-3

Using bulked segregant analysis (BSA) method (Michelmore et al. 1991), *REL2* gene is showed to link with inset and deletion (InDel) marker R10M40 on rice chromosome 10 (data not shown). A preliminary mapping of *REL2* was conducted by genotyping of 94 F_2_ mutant individuals with simple sequence repeat (SSR) markers near R10M40. Genetic mapping of the target gene revealed that *REL2* is linked with the SSR markers at the long arm of chromosome 10 (Fig. 4a)*.* For fine mapping of *REL2*, a series of InDel and single nucleotide polymorphism (SNP) markers were newly developed based on the genome sequence differences between Nipponbare and Kasalath (Fig. 4b, c). Genotyping of 882 F_2_ mutant individuals revealed that *REL2* is located between SNP markers SN22625 and SN22660, and co-segregated with SN22636 and SN22653 (Fig. 4c). Therefore, the *REL2* was mapped to a 35-kb genomic region by SN22625 and SN22660. According to the Rice Annotation Project Database, there are six genes annotated in the 35-kb region (Fig. 4d). A qRT-PCR analysis of all the genes in this region revealed that there is no difference at mRNA level between WT and *rel2* mutant (data not shown). We then sequenced all the genes of WT and *rel2* mutant with genomic DNA as template, and only found that *rel2* mutant has three sequence mutations in locus *Os10g0562700*, including a 4-bp deletion at 5’-UTR and two nucleotide substitutions at open reading frame (ORF) region (Fig. 4e). In fact, the mRNA level of *Os10g0562700* has no obvious difference between WT and *rel2* mutant (Additional file 2: Figure S2A, B), indicating that the 4-bp deletion in 5’-UTR in *rel2* mutant does not affect its transcription expression level. However, the mutation of two nucleotide substitutions in the ORF region causing amino acid substitutions (Cys^3^ to Tyr^3^, Val^14^ to Met^14^) should lead to loss of *REL2* function in the mutant. Therefore, the locus *Os10g0562700* should be the target gene, *REL2*. To confirm whether the *REL2* disruption resulted in the abnormal leaves, calli induced from *rel2* mutant seeds were transformed via *Agrobacterium tumefaciens* with *p35S-1301-REL2*, a construct with the full length of *REL2* ORF driven by a CaMV35S promoter. The positive plants were detected by GUS array and RT-PCR analysis. In order to know whether the mutant phenotype can be rescued, 23 independent transgenic lines were identified by observing the phenotype of leaves at early tillering stage. The results showed that rolling leaves of the transgenic plants are rescued and the phenotype of the leaves is just as the same to WT (Fig. 5a, b). Also, the results from RT-PCR analysis of transgenic plants showed that the mutant plants could be complemented by the over-expression of *REL2* (Fig. 5c), indicating that the normal REL2 protein expression restored the mutant phenotype. These results indicated that the mutant phenotype should be caused by the abnormal function of REL2 protein.

**Fig. 6:**
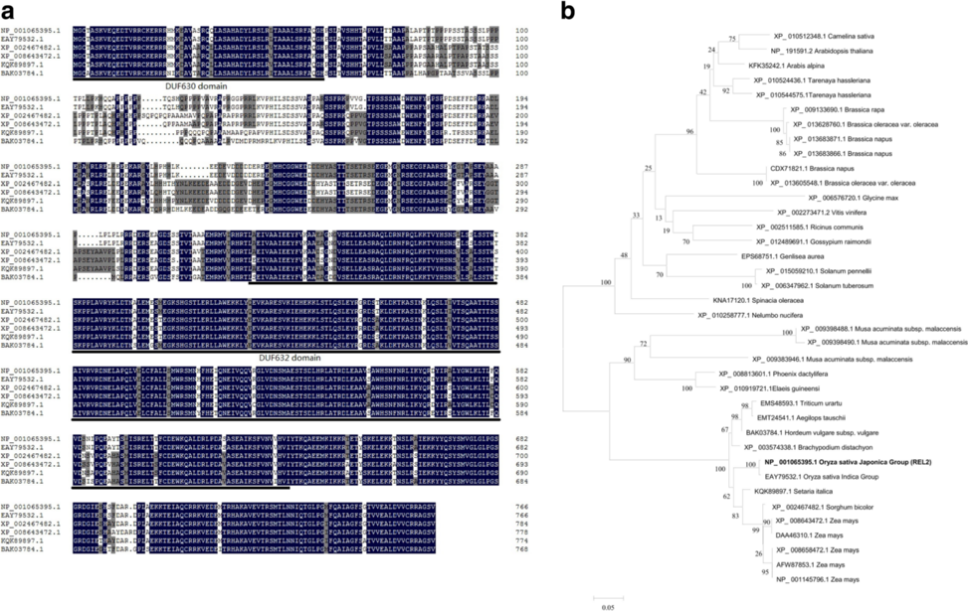
Multiple sequences alignment of REL2 with its highly similar protein sequences and phylogenic tree. **a** Multiple sequences alignment of *Oryza sativa Japonica Group* [GenBank accession number: NP_001065395.1, **REL2**], *Oryza sativa Indica Group* [GenBank accession number: EAY79532.1], *Sorghum bicolor* [GenBank accession number: XP_002467482.1], *Zea mays* [GenBank accession number: XP_008643472.1], *Setaria italic* [GenBank accession number: KQK89897.1] and *Hordeum vulgare subsp. vulgare* [GenBank accession number: BAK03784.1]. The amino acids showing identity are shaded *dark blue*, whereas similar amino acids are shaded *grey*. **b** Phylogenic tree of REL2 and its highly similar proteins. The protein sequences are displayed as GenBank accession number and species name

In order to get more information about the *REL2* gene, bioinformatics analysis was carried out. High similarity of protein sequences were selected for bioinformatics analysis. After that, a multiple sequences alignment was carried out and evolutionary tree was constructed. The results showed that the DUF630 and DUF632 domains in rice are highly conservative and always together and exhibit relatively close genetic relationship with the domains from *Setaria italic, Sorghum bicolor* and *Zea mays* while these two DUF domains were not close with those from *Arabidopsis thaliana* (Fig. 6a, b). However, all the protein sequences selected have not been studied in detail and their functions are unknown yet, so we concluded that *REL2* is a DUF domains contained unknown function protein.

### Expression Patterns of *REL2* and Subcellular Localization of the Protein

**Fig. 7:**
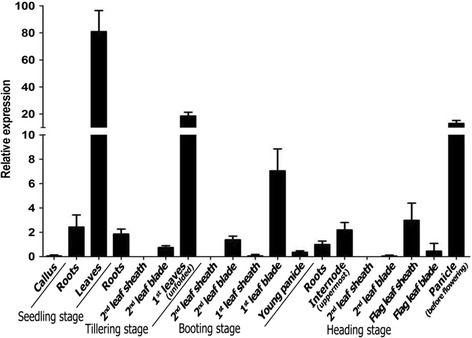
qRT-PCR analysis of the expression pattern of *REL2*. Total RNA was extracted from various tissues from callus and WT plants. Rice *Actin1* gene was used as a control. The expression of *REL2* in roots at heading stage was set to 1. The 1^st^ leaf blades or 2^nd^ leaf blade mean the position of leaf blades or leaf sheaths. From up to down of a rice plant, the first leaf blade or leaf sheath is the 1^st^ leaf blade or leaf sheath and the second leaf blade or leaf sheath is the 2^nd^ leaf blade or leaf sheath. The values are the mean ± SEM with three biological replicates

In order to investigate the expression pattern of *REL2*, total RNA was extracted from various tissues at different stages of wild-type rice. The expression level in roots at heading stage was set to 1. The results showed that the level of *REL2* transcription is highest in leaves at seedling stage. Also, the transcription level is relatively high in the 1^st^ leaf blades (the top leaf blades) at tillering stage, the 1^st^ leaf blades at booting stage and panicle before flowering at heading stage. However, there is no expression at the 2^nd^ leaf sheaths (the second leaf sheath from the top leaf sheath) at tillering, booting and heading stages. Meanwhile, the transcription level of *REL2* in other tissues is quite low (Fig. 7). These results suggested that *REL2* is dominantly expressed in the relatively younger leaf blades.

**Fig. 8:**
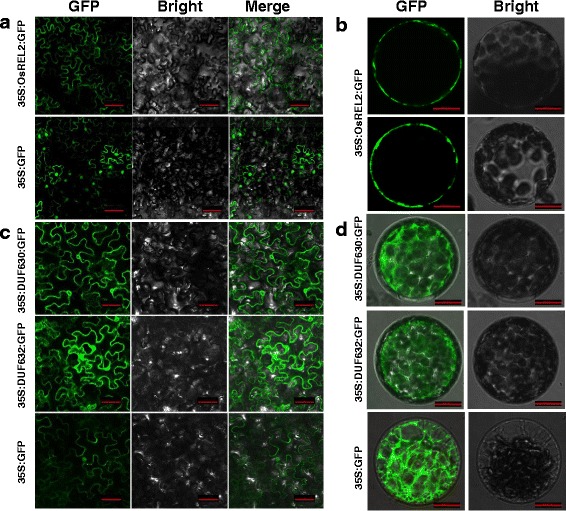
Subcellular localization of *REL2*. **a** Analysis of the subcellular localization of *REL2* using a tobacco transient transformation system in the cells of tobacco leaves. Bars = 100 μm. **b** Subcellular localization of *REL2* with tobacco protoplasts. Bars = 10 μm. **c** Subcellular localization of DUF630 and DUF632 in the cells of tobacco leaves. Bars = 50 μm. **d** Subcellular localization of DUF630 and DUF632 with tobacco protoplasts. Bars = 100 μm

To investigate the location of REL2 protein in cells, full length complementary DNA (cDNA) of *REL2* without stop condon was introduced into the *p35S-GFP* expression vector. To determine the roles of the two DUF domains in the location of REL2 protein, both DUF630 and DUF632 domain sequences were cloned into the *p35S-GFP* expression vector, respectively. The fused genes were injected into tobacco and the fluorescent signal was detected after transformation. As can be seen, the fluorescent signal from the infection with the full length cDNA of *REL2* was observed in the plasma membranes of the cells from both tobacco leaves and tobacco protoplasts (Fig. 8a, b), indicating that REL2 is a plasma membrane located protein. However, the two infections with the gene pieces of containing DUF630 and DUF632 domains separately, exhibited no specific localizations in the tobacco leaf cells and protoplasts, implying that both the two domains separated peptides have no plasma membrane localization in the cells (Fig. 8c, d).

### *rel2* Mutation Affects the Expression of Several Leaf Shape Regulation Genes

**Fig. 9:**
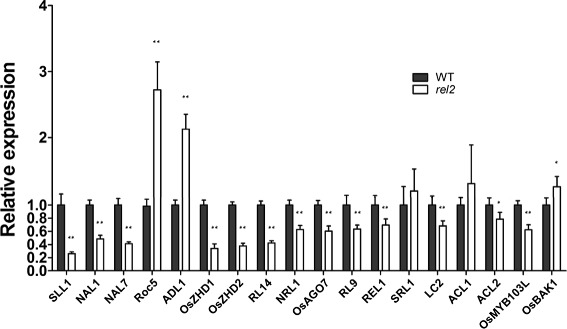
qRT-PCR analysis of the expression of genes related to leaf shape regulation in *rel2* mutant. Total RNA was extracted from leaves of WT and *rel2* mutant at seedling stage. Rice *Actin1* gene was used as a control. The values are the mean ± SEM with three biological replicates. *Double asterisk* (* *) indicates that the difference between the WT and *rel2* is statistically significant at *P* < 0.01. *Single asterisk* (*) indicates that the difference between the WT and *rel2* is statistically significant at *P* < 0.05

To determine the expression relationship of *REL2* with the leaf shape regulation associated genes, a total of eighteen genes were selected to be analyzed by qRT-PCR (Fig. 9). Total RNA was extracted from young leaves of both WT and *rel2* mutant at seedling stage. The results showed that *Rice outermost cell-specific gene 5* (*Roc5*) (homeodomain leucine zipper class IV gene) (Zou et al. 2011), *ADL1* (plant-specific calpain-like cysteine proteinase gene) (Hibara et al. 2009) and *OsBAK1* (a BR signaling related gene) (Li et al. 2009) are significantly up-regulated in *rel2* mutant. In contrast, genes called *SLL1* (a KANADI transcription factor) (Zhang et al. 2009), *NARROW LEAF 1* (*NAL1*) (a gene encoding a plant-specific protein affecting cell division) (Qi et al. 2008; Jiang et al. 2015), *NARROW LEAF 7* (*NAL7*) (a flavin-containing monooxygenase gene) (Fujino et al. 2008), *OsZHD1* and *OsZHD2* (zinc finger homeodomain class homeobox transcription factors) (Xu et al. 2014), *ROLLED LEAF 14* (*RL14*) (a 2OG-Fe (II) oxygenase family protein gene) (Fang et al. 2012), *NRL1* (a cellulose synthase-like D4 protein gene) (Wu et al. 2010), *OsAGO7* (an Argonatue family gene) (Shi et al. 2007), *RL9* (a GARP protein gene) (Yan et al. 2008), *REL1*(encoding a novel no known protein) (Chen et al. 2015), *LC2* (a vernalization insensitive 3-like protein gene) (Zhao et al. 2010), *ACL2* (a gene containing unknown conserved functional domains) (Li et al. 2010) and *OsMYB103L* (an R2R3-MYB transcription factor gene) (Yang et al. 2014) are down-regulated significantly in *rel2* mutant. These findings suggested that there might be a functional association between REL2 and those genes in regulating leaf morphological development of rice. By contrast, *SRL1* and *ACL1* have no expression changes in *rel2* mutant, indicating that the two genes may not have functional relationship with *REL2* (Fig. 9).

## Discussion

### The Leaf Rolling Phenotype of *rel2* should Result from the Abnormal Development of Bulliform Cells

There are several factors leading to leaf rolling in plants. Generally, leaf rolling is caused by the water loss of bulliform cells on adaxial epidermis in rice (O’Toole and Cruz 1980). This indicated that the number and area even the phenotype of bulliform cells may have effects on leaf rolling. Also, some other environment factors can affect leaf rolling such as high air temperature and strong sun light (Kidner and Timmermans 2007). Because the large and highly vacuolated vascular bundles bulliform cells on the adaxial epidermis of leaves participate in the regulation of leaf rolling (Xiang et al. 2012), uncovering the genes related to bulliform cells development is a necessary way to figure out the mechanism of leaf rolling. Until to now, genes associated with the development of bulliform cells have been studied. For example, *srl1* is involved in the regulation of leaf rolling and *srl1* mutant exhibits adaxially rolled leaves due to the increased numbers of bulliform cells at the adaxial cell layers. Further study showed that *SRL1* gene regulates leaf rolling by inhibiting the formation of bulliform cells through negatively regulating the expression of genes encoding vacuolar H^+^-ATPase subunits and H^+^-pyrophosphatase (Xiang et al. 2012). This mechanism is relatively clear and confirms that the number of bulliform cells is indeed to affect the leaf rolling. Also, the mutant of *ADL1*, *ACL1*, *NAL1*, *ROC5* and *OsZHD1* genes could lead to rolling leaves by changing the number and area of bulliform cells in adaxial leaf blades (Fujino et al. 2008; Hibara et al. 2009; Li et al. 2010; Zou et al. 2011; Xu et al. 2014). In the present study, the *rel2* mutant also shows changed phenotype of bulliform cells. The number of bulliform cells is obviously increased, but the size of middle bulliform cells is significantly decreased in leaf blades of *rel2* mutant as compared to WT. In addition, the *rel2* mutant leaf blades have reduced depth of bulliform cell region and the plant architecture is more erect by the straight leaves due to the leaf rolling (Fig. 1a, b). Even more, the expression patterns of *REL2* indicated that *REL2* has relatively higher expression in leaf blades (Fig. 7) which is consistent with the *rel2* mutant phenotype of leaf blades. The changes of bulliform cells in leaf blades may indicate that *REL2* might be related with the development of bulliform cells. The higher expression of *REL2* in panicle (before flowering) is also consistent with the changed traits such as seed shape, grains number per panicle. Although many studies showed that the leaf rolling can contribute to the photosynthetic efficiency (Yuan 1997; Zhang et al. 2009), the net photosynthetic rate of *rel2* is actually decreased as compared with that of WT (Fig. 2b), indicating a complicated mechanism involves in the functions of REL2. This needs to be further elucidated.

### DUF630 and DUF632 Domains may be Together to Implement their Function

As shown in Fig. 6a, the REL2 protein contains a DUF630 domain at the N-terminus and a DUF632 domain at the C-terminus. The DUF630 domain containing 59 amino acids was annotated that it was sometimes found at the N-terminus of putative plant bZIP protein and structural modeling suggested that this domain may bind nucleic acid. Meanwhile, the DUF632 domain containing 309 amino acids was described as a leucine zipper even though there was no experimental evidence supporting that (http://blast.ncbi.nlm.nih.gov/). However, the subcellular localization analysis of the entire protein sequence of REL2 in tobacco indicated that this protein is located on plasma membrane (Fig. 8a, b). But since no transmembrane domains were found in its animo acid sequence, REL2 may encode a plasma membrane anchored protein. Apart from this, the peptides containing DUF630 or DUF632 alone exhibit no special localization in tobacco cells (Fig. 8c, d). Considering the evolutionary conservation of DUF domains (Goodacre et al. 2014) and the co-evolution of DUF630 and DUF632, the results obtained here clearly indicated that the two DUF domains should be together to implement their functions. Since that all the protein sequences from different species were not studied in detail, our current study provides a primary reference for the function of DUF630 and DUF632 domains in plants.

### *REL2* may Regulate Leaf Shape by Coordination with Multiple Biological Factors

Multiple biological factors might participate in *REL2* controlled leaf rolling. It has been suggested that plant hormones play important roles in plant growth and development. For example, BR is vital for plant growth and development, especially in the regulation of leaf morphology and a rolled or erect leaf phenotype can always be observed by the BR-deficient mutants (Bishop and Yokoka 2001; Hong et al. 2004). For instance, the *rel1* mutant has rolled and erect leaves and a yeast two-hybrid screen experiment proved that *REL1* involves in the coordination of BR signaling transduction (Chen et al. 2015). However, the results from our leaf inclination experiment with BR showed that there may be no relationship between *REL2* gene and BR (data not shown). Another well known hormone which participates in plant growth regulation and development is auxin, acting as a signal for cell division, cell elongation and cell differentiation. Auxin also plays a key role in regulating the leaf primordum at the indeterminate SAM and cell growth in the development of leaves (Kepinski 2006; Tax and Durbak 2006; Fujino et al. 2008). In rice, *NAL1* and *NAL7* encode a putative trypsin-like serine/cysteine protease and a flavin-containing monooxygenase which displays sequence homology with *YUCCA,* respectively. Both of them participate in auxin regulation. *NAL1* affects vein patterning and polar auxin transport (PAT) and *NAL7* has an effect on auxin biosynthesis (Fujino et al. 2008; Qi et al. 2008; Cho et al. 2014). In the present study, we found that the transcription expression of both *NAL1* and *NAL7* is down-regulated obviously in the *rel2* mutant, indicating that the function of *REL2* gene in rice leaf rolling may be related to the auxin regulation also (Fig. 9).

Other biological factors might also participate in REL2-controled leaf rolling. It has been suggested that *SLL1* is involved in the development of sclerenchymatous cell on the abaxial side of rice leaves and *SLL1* deficiency leads to increased chlorophyll and photosynthesis (Zhang et al. 2009). In *rel2* mutant, *SLL1* was down-regulated obviously, indicating that *REL2* may have certain relationship with the sclerenchymatous development. In addition, the increased chlorophyll contents but decreased net photosynthetic rate of *rel2* mutant further implied that the functions of *REL2* in leaf rolling might be related to *SLL1. RL14* is a 2OG-Fe (II) oxygenase family protein gene which affects secondary cell wall formation in rice (Fang et al. 2012). Here, we found that its expression was also obviously down-regulated in the *rel2* mutant, suggesting that *REL2* may have some relationship with the lignin and cellulose biosynthesis for secondary cell wall formation, also. Moreover, three other genes, *ROC5*, *OsZHD1* and *OsZHD2*, were found to be up-regulated or down-regulated apparently in *rel2* mutant. These three genes were reported affecting the number, size and arrangement of bulliform cells in rice (Zou et al. 2011; Xu et al. 2014). Considering the phenotype of bulliform cells in *rel2* also changed significantly, it can be suspected that the functioning of *REL2* gene in the formation of bulliform cells might have somewhat linkages with the three genes. *ADL1* is a plant-specific calpain-like cysteine proteinase and was proposed to regulate the pattern formation of leaf and embryo in rice (Hibara et al. 2009). Up-regulated of *ADL1* in *rel2* mutant indicated that *REL2* might have function in the pattern formation and embryo, too. Overall, these results suggested that *REL2* is a pleiotropic gene and may participate in various biological processes with many other functional genes as discussed above.

To sum up, all of these results suggested that a complicated mechanism might be involved in the leaf rolling control in rice. The function of REL2 in this process needs further studies.

## Conclusions

A new leaf rolling controlling gene, named *REL2*, was identified in rice by using the map-based cloning strategy*.* This gene encodes a DUF630 and DUF632 domains containing protein and its loss of function led to rolling and erect leaves. The rolling leaves were caused by the abnormal development of bulliform cells and the functions of *REL2* might involve in multiple biological processes such as bulliform cell development, auxin synthesis and transport, secondary cell wall formation, etc. REL2 is the first discovered DUF domains contained protein which involves in the regulation of leaf morphology in plants.

## Methods

### Plant Materials and Growth Conditions

The *rel2* mutant with rolling leaves was isolated from the M_2_ generation of *japonica* rice cultivar Nipponbare by EMS treatment (rice seeds were treated with 0.4 % EMS for 8 h). The F_2_ mapping population was generated from a cross between mutant *rel2* and normal *indica* rice cultivar Kasalath. 882 *rel2* individuals were identified from the F_2_ mapping population in total. All plant materials were grown in the paddy field in Hanzhong, Shannxi province, China, in the summer of 2013 and 2014.

### Measurement of LEI and LRI

The plants at heading stage were used for the determination of the leaf erect index (LEI) and leaf rolling index (LRI). For testing LEI, Lsl (the distance between lamina joint and the tip of the leaf blade at stretched situation) and Lnl (the distance between lamina joint and the tip of the leaf blade at natural condition) were measured. For testing LRI, Lw (expand the leaf blade and determine the greatest width) and Ln (the natural distance of leaf blade margins at the same position) were measured. Then the LEI and LRI can be calculated by the following formulas (Shi et al. 2007):

$$ \mathrm{LEI}\ \left(\%\right) = {\mathrm{L}}_{\mathrm{nl}}/\ {\mathrm{L}}_{\mathrm{sl}}\times 100 $$

$$ \mathrm{L}\mathrm{R}\mathrm{I}\ \left(\%\right) = \left({\mathrm{L}}_{\mathrm{w}}\hbox{-}\ {\mathrm{L}}_{\mathrm{n}}\right)\ /\ {\mathrm{L}}_{\mathrm{w}}\times 100 $$

### Pigment and Net Photosynthetic Rate Measurement

0.2 g fresh leaves of rice in tillering stage were harvested to extract pigments and the contents of pigments were measured by a spectrophotometer according to the method described previously (Arnon 1949). Simply, leaves were cut into pieces and marinated in 95 % ethanol for 12 h under dark environment. Then, the pigments were collected by centrifugation and determined with a Spectrophotometer Evolution 300 (Thermo scientific, US) at 665, 649 nm, respectively. Three independent samples from different plants of WT and *rel2* were used for the analysis*.* Net photosynthetic rates and transpiration rates were measured at heading stage with the portable photosynthetic LCPRO_**+**_ instrument (ADC Bioscientific, UK) in the morning between 9:00 and 11:00. The measurement of net photosynthetic and transpiration rates of both groups of WT and *rel2* mutants was repeated at least three times on every different plant and at least five plants of each type plant were investigated.

### Histological Analysis

The flag leaves of WT and *rel2* were fixed in FAA (Formalin-Aceto-Alcohol) solution for 2 h. Leaf pieces with an area approximately 0.5 cm × 0.3 cm of WT and *rel2* plants at heading stage were hand-sectioned by using a razor blade. The sections of about 20-40 μm thickness were chosen to be examined by fluorescence microscopy DMIRB (Leica, Germany). Photographs were taken with a Leica light microscope and digital images were captured by using a DFC300 FX camera (Leica, Germany). At least three independent experiments were carried out for each analysis. Adobe photoshop CS4 software was used to calculate the depth and the number of bulliform cells (Bai et al. 2008).

### DNA Extraction and PCR Analysis

Rice genomic DNA was extracted from fresh leaves using modified CTAB (Hexadecyltrimethy Ammonium Bromide) method (Murray and Thompson 1980) at heading stage when significantly different phenotypes appeared. PCR reaction system was as follows: denaturation at 94 °C for 10 min; then followed by 32-35cycles of 94 °C for 30s, 53–58 °C for 30s, 72 °C for 30s, and the final step was extension for 5 min. After that, the PCR products were separated by 8 % polyacrylamide gel electrophoresis and the amplified bands were detected by silver staining.

### Map-based Cloning of *rel2*

In order to locate the position of the *rel2* on the rice chromosomes, 162 SSR (simple sequence repeat) markers and 37 InDel (insertion and deletion) markers were obtained from the rice GRAMENE database (http://www.gramene.org/) and (Shen et al. 2004). These markers were distributed evenly on rice 12 chromosomes and polymorphism between Nipponbare and Kasalath was displayed. Two pools (normal pool and mutant pool) were constructed by using equal amount of DNA of WT and F_2_ mutant with 10 individuals for each. The polymorphic markers screened in bulk segregant analysis (BSA) (Michelmore et al. 1991) were used for linkage analysis and molecular mapping. 94 mutant individuals from F_2_ were used for primary mapping. For fine mapping, a larger population with 882 mutant individuals from F_2_ was used. All the PCR products were analyzed by 8 % polyacrylamide gel electrophoresis.

### Phylogenetic Analysis

The full-length amino acid sequence of REL2 was downloaded from National Center for Biotechnology Information (NCBI) and its more than 50 % similarity protein sequences were obtained through a search of NCBI non-redundant protein sequences database (http://blast.ncbi.nlm.nih.gov/Blast.cgi). The alignment of amino acid sequences was carried out by using ClustalX2.0 (Jeanmougin et al. 1998). A neighbor-joining tree was generated by MEGA5.1 software by the bootstrap method with 1,000 replicates (Tamura et al. 2011).

### Development of New InDel and SNP Markers

To confirm the *rel2* at a smaller range on the rice chromosomes, new InDel and SNP markers were developed because of the lack of known markers. In order to develop new markers, the *japonica* rice cultivar Nipponbare genome sequences according to IRGSP (2005) were used as a query to do a BLAST against the whole genome sequences database of *indica* rice cultivar Kasalath (http://blast.ncbi.nlm.nih.gov/). Differences of more than two nucleotides were chosen to design InDel markers and single nucleotide polymorphisms were used to design SNP markers. All the new designed markers were validated by PCR analysis and polyacrylamide or agarose gel electrophoresis to acquire the polymorphic markers between Nipponbare and Kasalath. The new InDel and SNP markers were listed in Additional file 3: Table S1.

### Sequencing Analysis of the Candidate Region

To understand the possible functions of candidate genes, the annotation of candidate genes had been referring to the rice GRAMENE database (http://www.gramene.org) and the Rice Annotation Project Database (http://rapdb.dna.affrc.go.jp/viewer/gbrowse/build4/). Furthermore, six pairs of primers were synthesized to amplify the whole gene sequences of *Os10g0562100, Os10g0562200, Os10g0562500, Os10g0562550, Os10g0562600 and Os10g0562700* (Additional file 4: Table S2). Specific DNA fragments amplified by PCR from genomic DNA of WT and *rel2* were cloned into pMD19-T vector and then sequenced by Invitrogen Biotechnology Co., Ltd (Shanghai, China). To determine the mutant sites, the specific sequences were used to do a BLAST against the whole genome sequences Nipponbare (http://blast.ncbi.nlm.nih.gov/).

### Complementation of the *rel2* Mutant

For functional complementation of *rel2* mutant, the full length of WT cDNA of *REL2* was cloned into the binary *p35S-1301* vector, which contained a hygromycin resistance marker. Calli induced from *rel2* mutant seeds was used for transformation with *Agrobacterium tumefaciens* EHA105 carrying the *p35S-1301-REL2* plasmid. The positive plants were identified by GUS array. To determine whether the phenotype of *rel2* mutant was recovered by the over-expression of *REL2*, the leaves and expression of *REL2* of T_0_ transgenic lines were investigated.

### Subcellular Localization of *REL2*

Full length coding sequence of *REL2* with eliminated stop condon was cloned into *p35S-GFP* vector. Meanwhile, the full length protein sequence of REL2 was divided into two parts named DUF630 (1AA-314AA) and DUF632 (315AA-767AA), respectively. Both nucleotide sequences of DUF630 and DUF632 were cloned into *p35S-GFP* vector. The vector *p35S-GFP* without any pieces of *REL2* sequence was used as the control. All the vectors were transformed into *Agrobacterium tumefaciens* (EHA105). The infected *Agrobacterium tumefaciens* cells were harvested by centrifugation. Then a solution containing 10 mM MES (pH 5.6), 10 mM MgCl_2_ and 200 μM acetosyringone was applied to suspend the *Agrobacterium tumefaciens* cells to an optical density about 0.8–1.0 OD at 600 nm. After that, the 4-week old leaves of *Nicotiana benthamiana* were infiltrated with the *Agrobacterium tumefaciens* cells by using a needleless syringe. After growing for 3–4 days, the tobacco leaves were detected by a confocal microscope (AIR, Nikon, Japan). Also, the protoplasts from tobacco leaves which were infiltrated with the infected *Agrobacterium tumefaciens* cells were separated and observed with the same machine.

### RNA Isolation and Gene Expression Analysis

To investigate the expression patterns of *REL2*, total RNA was extracted from various tissues of WT plants at different developmental stages using RNAiso™ Plus (TaKaRa, Dalian) according to the manufacturer’s protocol. For analysis of *REL2* cDNA sequence in *rel2* mutant, total RNA was extracted from young leaves of WT and *rel2* mutant respectively. The extracted RNA was treated with RNase-free DNaseI to eliminate genomic DNA contamination. First strand cDNAs were synthesized from 2.5 μg total RNA using the M-MLV reverse transcriptase kit (Transgen biotech, Beijing). Both RT-PCR and qRT-PCR were used to analyze the expression level of *REL2*/*Os10g0562700* in WT and *rel2* mutant. The primers were listed in additional file 5: Table S3. qRT-PCR was carried out using the real-time PCR master mix (SYBR green, TaKaRa) with the CFX96 machine (Bio-Rad) following the manufacturer’s instructions. For analysis of *REL2* various tissues expression, a specific fragment was amplified by using primers 5’-TGATCATCGTGACTTCACAGGC-3’ and 5’-TCTACCAGACCACGGACTTGC-3’. Also, to determine the expression levels of some rolling-leaf genes (*SLL1*, *NAL1*, *NAL7*, *Roc5*, *ADL1*, *OsZHD1*, *OsZHD2*, *RL14, NRL1, AGO7, RL9, REL1, SRL1, LC2, ACL1, ACL2, OsMYB103L and OsBAK1*) in *rel2* mutants, qRT-PCR was carried out by using the specific primers listed in Additional file 5: Table S3. The rice *Actin1* gene specific primers 5’-CGTCAGCAACTGGGATGATATG-3’ and 5’-GTGTGGCTGACACCATCACCAG-3’ were used as an internal control.

## Abbreviations

BLAST, basic local alignment search tool; BSA, bulked segregant analysis; cDNA, complementary DNA; CTAB, hexadecyltrimethy ammonium bromide; DUF, domain of unknown function; EMS, ethyl methanesulfonate; FAA, formalin-aceto-alcohol; InDel, insert and deletion; IRGSP, international rice genome sequencing project; LEI, leaf erect index; LRI, leaf rolling index; mRNA, messenger RNA; NCBI, National Center for Biotechnology Information; ORF, open reading frame; REL, rolling and erect leaf; SAM, shoot apical meristem; SNP, single nucleotide polymorphism; SSR, simple sequence repeat

## Additional files

Additional file 1: Figure S1. Morphology of roots between WT and *rel2* mutant. A Phenotype comparison of roots between WT and *rel2* mutant. B Statistical analysis of the number of adventitious roots among WT and *rel2* mutant. The values are the mean ± SD (n = 10). Single asterisk (*) indicates that the difference between the WT and *rel2* is statistically significant at *P* < 0.05. (JPG 150 kb) Additional file 2: Figure S2. Analysis of *REL2* expression level in WT and *rel2* mutant. A RT-PCR analysis of *REL2* transcription expression level. B qRT-PCR analysis of *REL2* transcription expression level. The values are the mean ± SEM with three biological replicates. Rice *actin1* gene was used as a control. (JPG 36 kb) Additional file 3: Table S1. New developed InDel markers and SNP markers used for mapping. (DOCX 12 kb) Additional file 4: Table S2. Primers used for amplifying candidate genes. (DOCX 12 kb) Additional file 5: Table S3. Primers used for RT-PCR and qRT-PCR. (DOCX 14 kb)
