# Supplementary figures and images for: REL2, A Gene Encoding An Unknown Function Protein which Contains DUF630 and DUF632 Domains Controls Leaf Rolling in Rice
Source: Rice (N Y). 2016 Jul 29;9:37. doi: 10.1186/s12284-016-0105-6 (PMC4967057; doi:10.1186/s12284-016-0105-6)

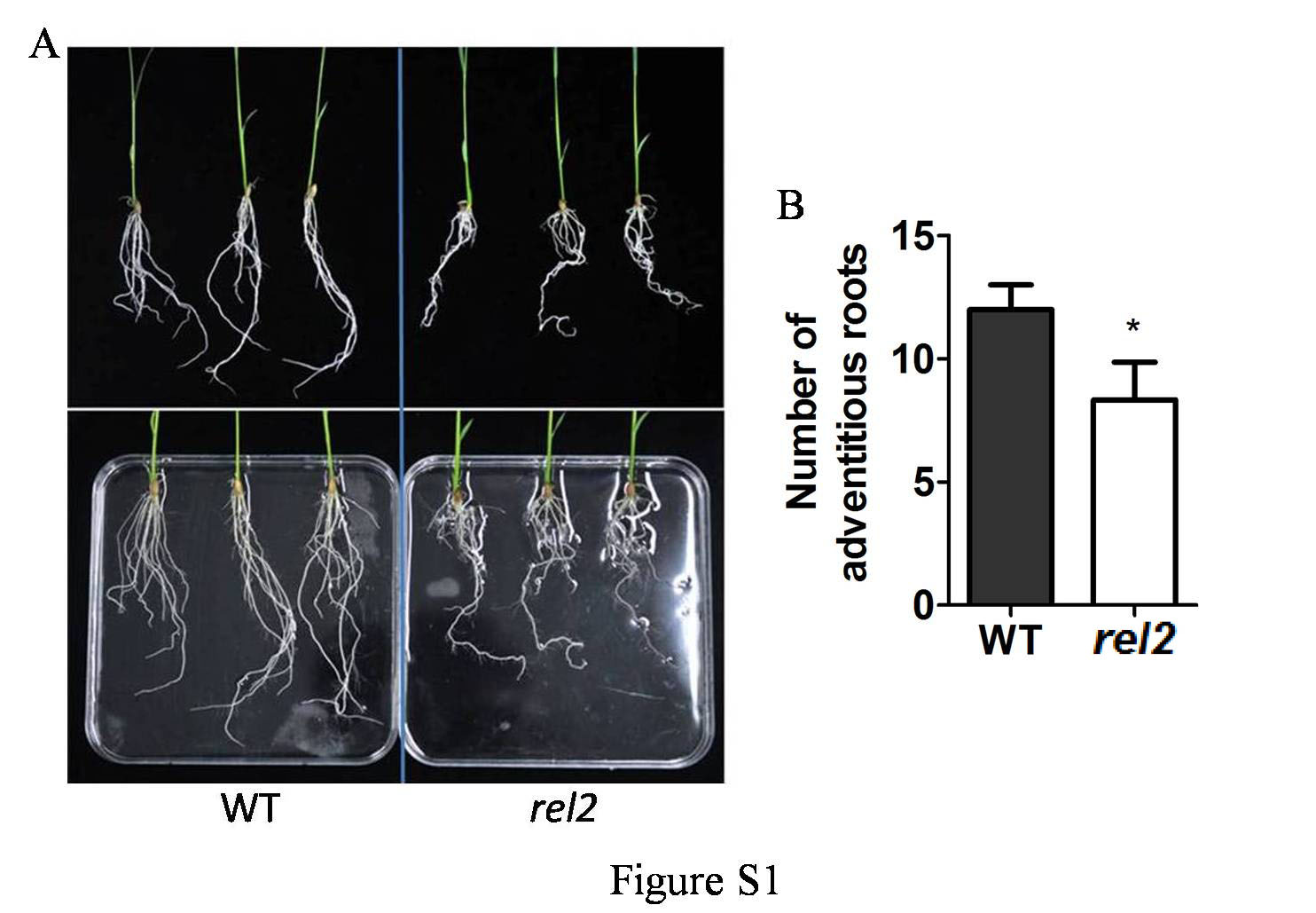

Supplement: Additional file 1: Figure S1. — Morphology of roots between WT and rel2 mutant. A Phenotype comparison of roots between WT and rel2 mutant. B Statistical analysis of the number of adventitious roots among WT and rel2 mutant. The values are the mean ± SD (n = 10). Single asterisk (*) indicates that the difference between the WT and rel2 is statistically significant at P < 0.05. (JPG 150 kb) [file 12284_2016_105_MOESM1_ESM.jpg]

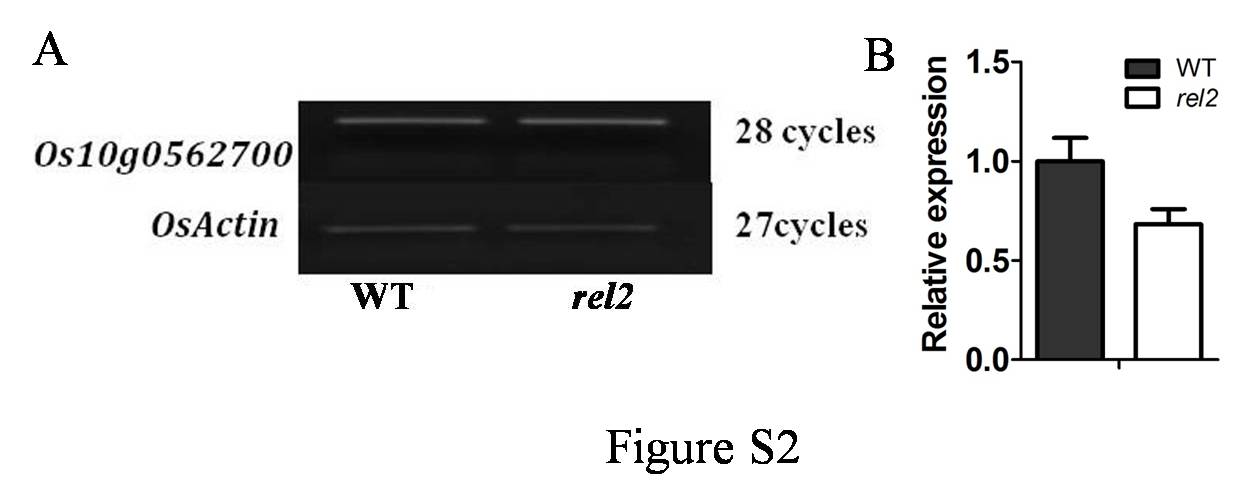

Supplement: Additional file 2: Figure S2. — Analysis of REL2 expression level in WT and rel2 mutant. A RT-PCR analysis of REL2 transcription expression level. B qRT-PCR analysis of REL2 transcription expression level. The values are the mean ± SEM with three biological replicates. Rice actin1 gene was used as a control. (JPG 36 kb) [file 12284_2016_105_MOESM2_ESM.jpg]
