# Supplementary material for: REL2, A Gene Encoding An Unknown Function Protein which Contains DUF630 and DUF632 Domains Controls Leaf Rolling in Rice
Source: Rice (N Y). 2016 Jul 29;9:37. doi: 10.1186/s12284-016-0105-6 (PMC4967057; doi:10.1186/s12284-016-0105-6)
Supplement: Additional file 3: Table S1. — New developed InDel markers and SNP markers used for mapping. (DOCX 12 kb) [file 12284_2016_105_MOESM3_ESM.docx]

| Primer name | Forward:5’ to 3’ | Reverse:5’ to 3’ |
| --- | --- | --- |
| ID22601 | ATCTCATGGGCCAAGCTATC | CCATACTTAGCTTCCTCCACAA |
| ID22728 | TGGCTTCCATCCTTTCTTATC | CAAATTTACTGGAGACGGGTT |
| SN22625 | TTATAAGAGTTATGAAGTCACAACAGATTTATACGG/ TTATAAGAGTTATGAAGTCACAACAGATTTATGCCA | TAATAACACAACATGTGCACATCCTCGAATG |
| SN22636 | GGCGAGTGGCGGAGGTATCAGA/GGCGAGTGGCGGAGGTATCATG | GCCCTCGTTGTCGCAGACGG |
| SN22653 | CCATCACAAGCCACACCACATCATTATCT/CCATCACAAGCCACACCACATCATTAGAA | TTTCATGCCCAAGTTGTGAGATGATTTGT |
| SN22670 | GAAAAGACTAGCTAGGCGCATGGAGTAGTAATG/CGAAAAGACTAGCTAGGCGCATGGAGTAGTAATA | TGTTGATCAAAATATGCCAATCTAAAGTTAAAACTG |
| SN22673 | TGAGGAACTAATATGTTGTCTGTTCCAATGGC/GTTGAGGAACTAATATGTTGTCTGTTCCAATGGT | TCCATAAGTAAACTAGCAAACAAATGCAGCATG |

**Table S1**

New developed InDel markers and SNP markers used for mapping
