# Supplementary material for: REL2, A Gene Encoding An Unknown Function Protein which Contains DUF630 and DUF632 Domains Controls Leaf Rolling in Rice
Source: Rice (N Y). 2016 Jul 29;9:37. doi: 10.1186/s12284-016-0105-6 (PMC4967057; doi:10.1186/s12284-016-0105-6)
Supplement: Additional file 4: Table S2. — Primers used for amplifying candidate genes. (DOCX 12 kb) [file 12284_2016_105_MOESM4_ESM.docx]

**Table S2**

Primers used for amplifying candidate genes.

| Gene name | Forward:5’ to 3’ | Reverse:5’ to 3’ |
| --- | --- | --- |
| *Os10g0562100* | GAGCCCAACAATAATAATCCCC | CTGCTGAAAGAAACGCCACAT |
| *Os10g0562200* | CATCTGCTGGAACTGCTGCTT | GACTCAACCGCATCACCTCT |
| *Os10g0562500* | CACATACCCCCTTTGACCGAT | GGCATTCACATTTTGATACGC |
| *Os10g0562550* | CTTGCTAGAAGAACTCAAAG | TTTGCAGGTTCTACGCC |
| *Os10g0562600* | CCAGAGACACATAAGGGCAATA | GAATTTAACGGTCAACTAGCGG |
| *Os10g0562700* | CAGCATTTGACTAATCCCTTCG | CCGCATAGTCCTACATGGTCAG |
