# Supplementary material for: REL2, A Gene Encoding An Unknown Function Protein which Contains DUF630 and DUF632 Domains Controls Leaf Rolling in Rice
Source: Rice (N Y). 2016 Jul 29;9:37. doi: 10.1186/s12284-016-0105-6 (PMC4967057; doi:10.1186/s12284-016-0105-6)
Supplement: Additional file 5: Table S3. — Primers used for RT-PCR and qRT-PCR. (DOCX 14 kb) [file 12284_2016_105_MOESM5_ESM.docx]

**Table S3**

Primers used for RT-PCR and qRT-PCR.

| Gene name | Forward:5’ to 3’ | Reverse:5’ to 3’ |
| --- | --- | --- |
| RT*-REL2* | TGTCATCTACACTAAGCAGGCA | TTTATCTACAACCAAAATGACCA |
| qRT*-REL2* | TGATCATCGTGACTTCACAGGC | TCTACCAGACCACGGACTTGC |
| *SLL1* | CAGCTCGCAGGTGTCCAA | CCTCCCTAGAGTGAACTCGAGACT |
| *NAL1* | TGCAGTGTCCGCTCAATAGC | GACCAGAGCTTCTGCCAACTTT |
| *NAL7* | CAAGAACATCACCGGCAAGA | CGATTTGATCAAGGACCATGCT |
| *Roc5* | CGCAAGAGGAAGAAGCGATAC | GCTCCAGTTGCGTCTTCATC |
| *ADL1* | ATCTGGCTTCTTCTTTGGGG | TGCCCCTTCCTTCGAAAACC |
| *OsZHD1* | CGGACCCCGGTATGGTAG | CGAGAACGAATGCTCTCTCAG |
| *OsZHD2* | CCGTGCAGCAGTTCTGCGA | CAGGGTGTGCTTGTTGTTGTG |
| *RL14* | CTCTTTCAGGCATTCCATTGATG | CAACACCTTGTCAGCTTTCAAGC |
| *NRL1* | TCAGTAGTGTAGTGGTGTCGAGTTCA | GCACTCCTTCATGTGAGCTTCA |
| *OsAGO7* | CCGCATCCCCTTGATGATT | GGCCAATTCATGCTTGCAA |
| *RL9* | ATTCTTGCAACATGGACGCC | CATTAGCCTCTGTGATTGCC |
| *REL1* | CAACGGCAAGAGTTCCAAGCT | CTCCGACGACACGCACGA |
| *SRL1* | CCCAATTCCTTGGTGACAAGT | TTGCATGAAGAAGACGATGCT |
| *LC2* | AGCATCAGCTTTGGACGAGGA | CAGTTGGTGGAATAGAGCCAGAAT |
| *ACL1* | CTGAAGCTGAACCTCTCGCTG | GGAGCATGACGTAGATGAAGCAG |
| *ACL2* | CTCGTGCCTGTCGTCGGAG | TCGGCGAGCATGACGTAGAG |
| *OsMYB103L* | CTTAGAAGATGGCCAAACAGCC | TGGCCTCCAAGTTGGATGAT |
| *OsBAK1* | GAGTTGATCTTGGGAATGCTGC | CACTAGGTATCGTTCCGCTTATGTT |
